# Rats subject to extracorporeal membrane oxygenation have improved cardiac function following anticoagulation and reversal with factor IXa aptamer-antidote oligonucleotide pair

**DOI:** 10.1097/CP9.0000000000000122

**Published:** 2025-06-24

**Authors:** Shahid M. Nimjee, Fellery de Lange, George A. Pitoc, Bruce A. Sullenger

**Affiliations:** 1 Department of Neurosurgery, The Ohio State University Medical Center, Columbus, Ohio 43210, USA.; 2 Department of Intensive Care, Medical Center Leeuwarden, Leeuwarden 8901 BR, The Netherlands.; 3 Department of Surgery, Duke University Medical Center, Durham, North Carolina 27710, USA.

**Keywords:** Heparin, Aptamer, Anticoagulant, Extracorporeal membrane oxygenation, Cardiopulmonary bypass

## Abstract

**Background and purpose:**

Unfractionated heparin (UFH) is the most commonly utilized rapid-onset anticoagulant, valued for its potency and reversibility with protamine. However, UFH and protamine are associated with significant side effects, including increased morbidity and mortality, and concerns about sustainability due to the environmental impact of large-scale pig farming for heparin production. This study evaluates an alternative anticoagulant strategy using a factor IXa (FIXa) aptamer paired with a matched oligonucleotide antidote, comparing its efficacy and safety to heparin-protamine in a rat extracorporeal membrane oxygenation (ECMO) model.

**Methods:**

Twenty-four Sprague-Dawley rats were randomized into two groups: one receiving heparin (600 IU/kg) and protamine (1 mg/100 IU heparin), and the other receiving a cholesterol-modified FIXa aptamer (10 mg/kg) and its antidote (50 mg/kg). Coagulation parameters, platelet counts, inflammatory markers, cardiac function, and histopathology were assessed during and after 60 minutes of ECMO.

**Results:**

The FIXa aptamer effectively maintained circuit patency without clot formation, comparable to heparin. The antidote rapidly reversed the aptamer’s anticoagulant activity, similar to protamine’s reversal of heparin. Notably, the aptamer-antidote group demonstrated superior outcomes, including improved mean arterial pressure (58 ± 6 mmHg vs. 54 ± 3 mmHg at 30 minutes; 59 ± 8 mmHg vs. 51 ± 5 mmHg at 3 hours post-ECMO) and cardiac function (shortening fraction: 60 ± 16% vs. 42 ± 8%; P = 0.01). Additionally, the aptamer group exhibited better platelet preservation (platelet count decrease: −288,000 ± 121,000/μL vs. −404,000 ± 89,000/μL; P = 0.03). Inflammatory profiles were similar between groups, except for a transient increase in interleukins 10 (IL-10) in the aptamer group. Histopathological analysis revealed no significant differences in myocardial lesions.

**Conclusions:**

The antidote-controlled anti-FIXa aptamer represents an alternative anticoagulant strategy that may prove useful for managing patients with a history of heparin-induced thrombocytopenia (HIT) and myocardial dysfunction associated with protamine administration.

## INTRODUCTION

Across all clinical subspecialties, unfractionated heparin (UFH) is the most commonly used rapid-onset intravenous anticoagulant^[1–2]^. This fact especially holds true in cardiology and cardiac surgery. Recently, clinicians have begun to reevaluate their “historical” first choice for anticoagulation therapy, and new anticoagulant drugs are being developed and evaluated in experimental settings. This change of view has resulted from the increasingly recognized significant side effects associated with heparin and its antidote protamine. UFH indirectly inhibits thrombin by acting as a cofactor for antithrombin III to bind to the active site of thrombin, inactivating it^[3]^; this process can deplete antithrombin III and result in heparin resistance^[4]^. Moreover, UFH has no effect on fibrin-bound thrombin^[5]^. Finally, UFH is a natural product, and the world’s yearly supply requires over 1 billion pigs to produce. Thus, the sustainability of heparin has recently been called into question given the greenhouse gases associated with its production^[6]^.

Both heparin and its antidote protamine have several side effects that can limit their clinical utility. Heparin binds to platelet factor 4 (PF4) on the surface of platelets^[7–8]^. This heparin–PF4 complex can elicit antibodies in patients, and these antibodies can lead to severe clinical complications. Moreover, it has been observed that increased anti-heparin–PF4 antibody levels are a predictor of adverse outcome in cardiac surgery, including major complications, extended hospital stay, and increased mortality^[9–10]^. Anti-heparin–PF4 antibodies also can cause heparin-induced thrombocytopenia (HIT) defined as a platelet count less than 150,000 per microliter, which affects 2% to 4% of patients^[7]^. Thus, with approximately 10 million patients receiving heparin annually in the United States^[1]^, tens of thousands of individuals are affected by HIT per year in the USA alone^[11]^. Of those, 30% will go on to develop HIT with thrombosis (HITT), which has a significant 30-day mortality rate^[8,12]^. Administration of protamine is also associated with clinical complications. Many individuals can have an allergic reaction to protamine, which is derived from salmon sperm^[13]^.

We previously reported a new class of antidote controllable anticoagulants based upon aptamers and matched oligonucleotide antidotes^[14–17]^. Aptamers are single-stranded oligonucleotides that fold into a specific three-dimensional structure enabling them to directly bind to and inhibit a protein target such as the coagulation factors (F)IX, FX, FXI, and FXII^[16,18–22]^. Advantageous properties of this class of drug agents include high affinity and specificity for their targets, low immunogenicity, modifiable bioavailability, and the ability to rationally design antidote oligonucleotides to reverse their activity^[19,23–24]^. Moreover, aptamers can be manufactured without requiring large animal farming for production.

Herein, we studied and compared clinically relevant clotting parameters, platelet count, cytokine release, short-term histopathological impact, and physiologic parameters of the factor IXa (FIXa) aptamer-oligonucleotide antidote to heparin-protamine in a rat extracorporeal membrane oxygenation (ECMO) circuit to mimic extracorporeal blood circulation conditions encountered during cardiopulmonary bypass (CPB) surgery.

## METHODS

The study was approved by the Duke University Institutional Animal Care and Use Committee (Approval #A272-04-10; 10-28-2004) and all procedures met the United States National Institutes of Health (NIH) ethical guidelines for animal care.

### Animals and groups

Twenty-four male 425 to 450 g Sprague-Dawley rats (Charles River Labs, Wilmington, MA, USA) were used for this study. They were housed three per cage under 12-hour light-dark cycle conditions with food and water available *ad libitum*. They were randomly assigned to one of two treatment groups (n = 10 per group); the first group received heparin 600 IU/kg before ECMO/CPB and protamine 1 mg per 100 IU of porcine heparin at the end of CPB and the other group received 10 mg/kg of the cholesterol-modified version of the FIXa aptamer before the start of ECMO/CPB and 50 mg/kg antidote oligonucleotide at the cessation of ECMO/CPB. Both drugs were diluted in saline to a volume of 0.3 mL. The experimenter performing the surgery, ECMO/CPB, echocardiography, and organ harvest was blinded as to the anticoagulant regiment employed in the animals.

### Anesthesia, surgical preparation, and CPB

After a 12-hour fast, anesthesia was induced with 5% isoflurane in 50% O_2_ in a plastic induction box. After orotracheal intubation with a 14 G catheter (Insyte BD Medical, Sandy, UT, USA), the animals were mechanically ventilated to a maximum airway pressure of 18 mmH_2_O (Harvard Model 687; Harvard Apparatus, Holliston, MA, USA), and an arterial carbon dioxide tension between 35 and 45 mmHg was maintained. During the surgical preparation, anesthesia was maintained with 2% to 2.5% isoflurane. A needle thermistor was inserted in the left temporal muscle adjacent to the skull to measure pericranial temperature. Temperature was maintained between 36.5°C and 37.0°C with both a forced-air and surface heating system.

**Figure 1.**
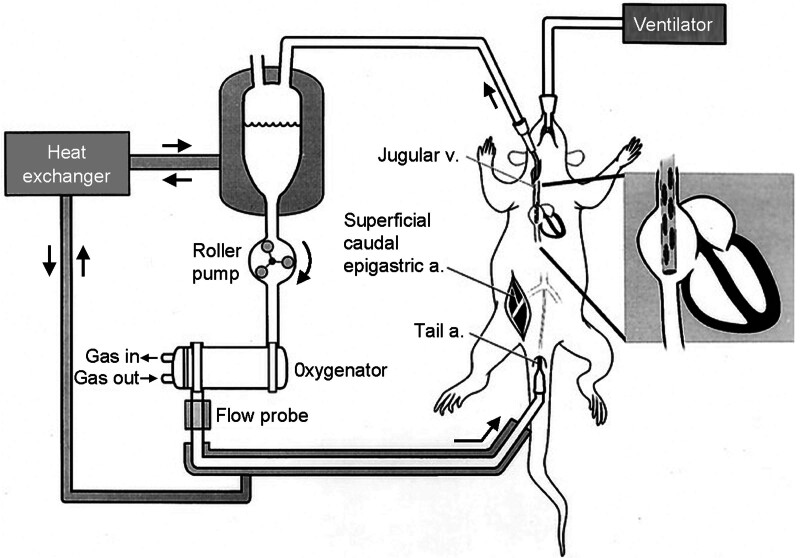
**A schematic representation of the rat extracorporeal membrane oxygenation circuit.** Adapted from Cao *et al*.^[24]^

As illustrated in Figure 1, the rat ECMO/CPB model used in this experiment was based upon that originally described by Mackensen *et al*.^[25]^ Briefly, arterial blood pressure was monitored via the superficial caudal epigastric artery, which was cannulated with polyethylene tubing (PE-10 Intramedic Tubing; Becton-Dickinson, Sparks, MD, USA). The superficial epigastric vein was cannulated with a 24 G catheter (Insyte BD Medical) and used solely for administration of drug and antidote. Further preparation consisted of cannulation of the tail artery with a 20 G catheter (Insyte BD Medical), which later served as arterial inflow for the CPB circuit. The full dose of porcine heparin (600 IU/kg) or aptamer 10 mg/kg was administered. The animal then received 5 μg of fentanyl for analgesia. A modified multi-orifice 4.5 French pediatric catheter (modified Desilets-Hoffman Catheter; Cook, Bloomington, IN, USA) was advanced through the right external jugular vein into the right heart for venous outflow.

The ECMO/CPB circuit consisted of a specifically designed 8 mL Plexiglas^®^ venous reservoir, a roller pump (Masterflex^®^; Cole-Parmer Instrument Co., Vernon Hills, IL, USA) and a custom-designed small-volume oxygenator^[10]^. The 4-mL priming volume oxygenator was built of two Plexiglas^®^ shells (12.8 cm × 12.8 cm × 2.7 cm) that carry a sterile, disposable three-layer artificial diffusion membrane, made with hollow polypropylene fibers (Jostra AG, Hirrlingen, Germany) glued together in a crosswise fashion. The surface area available for gas exchange was 558 cm^2^. To prevent excessive heat loss, one of the shells had an integrated heat exchanger. An in-line flow probe (2N806 probe and T208 flowmeter; Transonics Systems Inc., Ithaca, NY, USA) was used to measure blood flow continuously. The whole circuit was primed with 10 mL of 6% hetastarch (Hextend, Hospira Inc, Lake Forest, IL, USA) and 0.2 mg of pancuronium. The dilutional effect of the prime caused the hematocrit to be in the 0.22 to 0.24 range during CPB.

All parts were connected through silicone tubing, which was disposed of after every bypass run. During ECMO/CPB, ventilation was discontinued, and isoflurane 0.2% to 1% in 100% O_2_ was administered through the oxygenator. ECMO/CPB with a flow rate of 150 mL·kg^−1^·min^−1^, adjusted to maximize flow and to maintain a minimal venous reservoir blood level, was carried out for 60 minutes. Mean arterial pressure was kept between 50 and 60 mmHg. At 30 minutes of CPB, a repeated dose of 0.2 mg of pancuronium was added to the circuit. After 60 minutes of CPB, ventilation was reinitiated, and CPB was discontinued, and the jugular venous cannula was removed. Immediately thereafter, protamine or an antidote oligonucleotide was administered. The animals remained ventilated for another 3 hours. After the last blood sample was obtained at the 3-hour time point, the heart was harvested from each study animal and preserved in 10% formalin.

### Activated clotting time and activated partial thromboplastin time

Coagulation assays were performed at the following time points: (1) Pre-ECMO/CPB: beginning of the experiment, just before the start of ECMO/CPB, (2) ECMO/CPB: 30-minute intervals, (3) Post-ECMO/CPB: 20 and 60 minutes post-CPB, using a Hemochron^®^ Jr. Signature Whole Blood Microcoagulation system (International Technidyne Corporation, New Albany, NY, USA). The instrument was calibrated and used in this study similar to clinical studies^[26]^. We inserted an activated partial thromboplastin time (aPTT) cuvette into the instrument, and added whole blood to the reservoir (~50 μL). Results are display as plasma equivalents for aPTT performed upon duplicate samples.

### Blood gas analysis, interleukins, and platelets

Blood gas analysis was performed before the start of ECMO/CPB, at 30 and 60 minutes of ECMO/CPB, and at 1, 2, and 3-hour post-ECMO/CPB, using IL GEM Premier 3000 blood gas analyzer (Global Medical Instrumentation, Ramsey, MI, USA). Blood samples for interleukins 1β (IL-1β), interleukins 6 (IL-6), interleukins 10 (IL-10), tumor necrosis factor alpha (TNF-α), and thrombin-antithrombin (TAT) analysis were collected from the tail artery before the start of the experiment, during ECMO/CPB at intervals of 30 minutes, and post-ECMO/CPB at 1, 2, and 3 hours. Samples were immediately centrifuged at 4°C and the serum frozen and stored at −80°C for analysis. Serum TNF-α, IL-1β, IL-6, and IL-10 were analyzed by multiplexed sandwich enzyme-linked immunosorbent assay (ELISA) microtitre plate following manufacturer’s instructions (Endogen^®^ - Search Light™, Inc., Woburn, MA, USA). Results were expressed as pg/mL, and sensitivity for detection was 6.2 pg/mL for TNF-α, 12.6 pg/mL for IL-1β, 12.6 pg/mL for IL-6, and 3.1 pg/mL for IL-10.

### Platelet count

Blood for platelet counts was collected at the start of ECMO/CPB and 3-hour post-ECMO/CPB. Counts were performed using the Cell-Dyn 3700 (Abbott Diagnostics, Abbott Park, IL, USA).

### Pathological analysis

At the end of each experiment, the hearts were harvested for histopathological analysis. They were fixed in 10% formalin, placed in a cassette, and sent to clinical pathology for evaluation^[27]^. Samples were coded such that the pathologist performing the analysis was blinded to the anticoagulant used in the evaluated animal. Heart histology was analyzed by the same pathologist after samples were stained with hematoxylin and eosin. Myocardial contraction bands were visualized, and the associated lesions induced during CPB/ECMO were quantified for the entire organ by slicing it into sections (size is 1 × 1.5 cm) and followed by analyses as previously described^[28]^.

### Echocardiography

Three hours after the cessation of ECMO/CPB, the shortening fraction (SF) was estimated using ultrasonography (Sonosite 180 with 10-5 MHz broadband linear array transducer, Bothell, WA, USA) under 1.5% isoflurane in all rats. A transthoracic cross-sectional view of the left ventricle was obtained at the papillary level. Rat heart rates remained >200 bpm during the entire analysis. The end-diastolic diameter (EDD) and end-systolic diameter (ESD) were measured, and the SF was calculated (EDD − ESD/EDD × 100). All measurements were done using three different loops and averaged. The loops were reassessed by an investigator blinded to treatment designation.

### Statistical analysis

Physiological parameters, aPTT, and activated clotting time (ACT) during surgery and the postoperative period were analyzed using the Mann-Whitney *U* test. As the aPTT and ACT values max out the assay and do not have a clear cutoff value from the machine, there is no normality; therefore, we utilized the Mann-Whitney *U* test for statistical analysis. IL levels were analyzed using analysis of variance (ANOVA). Statistical analysis of the histological samples was performed using the Wilcoxon rank-sum procedure, χ^2^, and Cochran-Mantel-Haenszel (CMH). P values <0.05 were considered significant, and GraphPad PRISM 10 (San Diego, California, USA) was utilized for statistical analyses.

## RESULTS

### FIXa aptamer maintains patency of rat ECMO/CPB circuit

**Figure 2.**
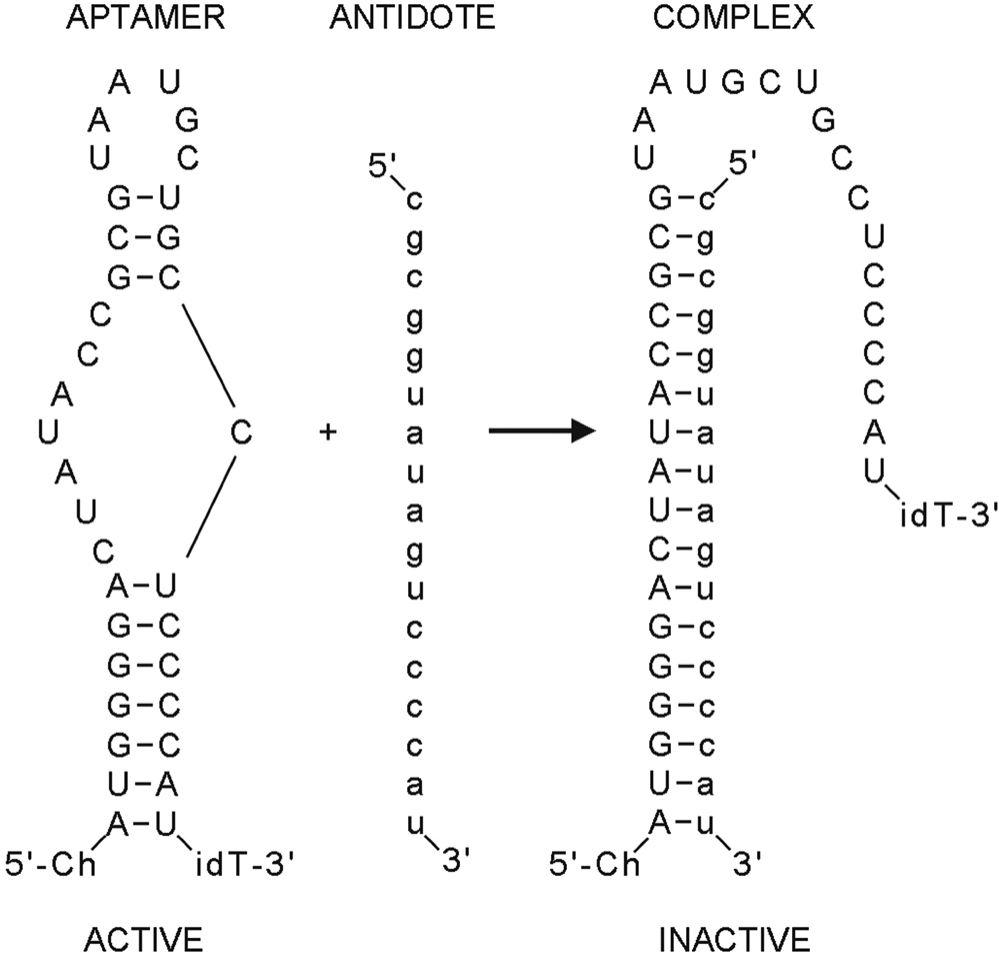
**Depiction of factor (F)IXa aptamer sequence and secondary structure and antidote oligonucleotide sequence.** The factor IX aptamer (left) is shown in capitals. The pyrimidines in the aptamer are modified with 2’F-nucleotides while the purines are composed of 2’OH-nucleotides. The 5’-end of the aptamer contains a cholesterol, and the 3’-end is blocked by an idT. The antidote oligonucleotide (middle) is shown in lower case and is comprised of 2’OMe nucleotides. It binds to the active aptamer *via* standard base-pairs to form an inactive complex (right). idT: inverted deoxy-thymidine.

To determine if the FIXa aptamer could effectively prevent blood clot formation in the rat ECMO/CPB circuit model, we established two groups: one treated with heparin and the other treated with the cholesterol-modified version of the FIXa aptamer (n = 10 per group) (Figure 2). The circuit remained patent throughout bypass in both the heparin- and aptamer-treated animals. Gross examination of the reservoir, tubing, and oxygenator revealed no evidence of thrombi.

### Enhanced post-reversal function in aptamer-antidote vs. heparin-protamine group

To determine if physiological differences existed between the groups treated with heparin-protamine compared to aptamer-antidote, we measured the weight, hematocrit, pericranial temperature, CPB flow, mean arterial pressure (MAP), pH, arterial partial pressures of carbon dioxide, oxygen, and bicarbonate ion. The results of most factors studied were similar in both groups (Table 1). However, an improved MAP of the aptamer-antidote-treated group at the 30-minute interval during ECMO/CPB and 3 hours post-ECMO/CPB was observed. At the 30-minute interval, the aptamer-treated group was 58 ± 6 mmHg compared to 54 ± 3 mmHg in the heparin-treated group (P = 0.04). At 3 hours post-ECMO/CPB, the aptamer-treated group was 59 ± 8 mmHg compared to 51 ± 5 mmHg in the heparin-treated group (P = 0.01).

**Table 1.** Physiological parameters in the two groups at different timepoints

Variables	Baseline	ECMO/CPB		1 h post	2 h post	3 h post
		30 min	60 min			
Weight, g						
Heparin	446 (17)	-	-	-	-	-
Aptamer	444 (22)	-	-	-	-	-
Hematocrit, %						
Heparin	36.3 (2.2)	24.3 (1.8)	24.2 (1.7)	22.7 (1.4)	21.0 (1.7)	20.3 (1.4)
Aptamer	36.4 (1.4)	24.2 (1.4)	23.9 (1.3)	22.8 (1.5)	21.3 (1.6)	19.6 (2.0)
Pericranial temperature, °C						
Heparin	36.3 (0.5)	35.3 (0.3)	35.4 (0.3)	36.2 (0.5)	36.1 (0.9)	36.3 (0.5)
Aptamer	36.4 (0.3)	35.2 (0.2)	35.2 (0.2)	36.2 (0.7)	36.6 (0.2)	36.5 (0.3)
CPB flow, mL/min						
Heparin	-	54 (3)	54 (5)	-	-	-
Aptamer	-	58 (6)^*^	58 (4)	-	-	-
MAP, mmHg						
Heparin	59 (7)	56 (12)	68 (15)	74 (10)	62 (14)	51 (5)
Aptamer	56 (5)	52 (14)	67 (9)	64 (15)	65 (10)	59 (8)^*^
pH						
Heparin	7.49 (0.06)	7.45 (0.04)	7.45 (0.04)	7.52 (0.05)	7.49 (0.05)	7.52 (0.07)
Aptamer	7.48 (0.03)	7.44 (0.06)	7.44 (0.03)	7.45 (0.12)	7.50 (0.03)	7.49 (0.09)
PaCO_2_, mmHg						
Heparin	39 (4)	42 (4)	42 (4)	37 (6)	36 (3)	29 (6)
Aptamer	39 (3)	43 (7)	44 (4)	39 (4)	36 (3)	31 (6)
PaO_2_, mmHg						
Heparin	128 (32)	496 (77)	507 (90)	356 (80)	368 (101)	388 (105)
Aptamer	148 (40)	522 (53)	534 (60)	363 (96)	390 (117)	405 (139)
HCO_3_ˉ, mmHg						
Heparin	29.3 (1.2)	28.6 (1.4)	29.3 (2.0)	30.2 (2.3)	27.5 (3.4)	24.5 (3.8)
Aptamer	28.7 (1.2)	29.1 (1.1)	29.8 (1.1)	28.8 (2.1)	27.7 (3.3)	24.6 (3.9)

Data are presented as mean ± SD.

* P < 0.05 compared to heparin group.

CPB: cardiopulmonary bypass; ECMO/CPB: extracorporeal membrane oxygenation/cardiopulmonary bypass; MAP: mean arterial pressure; PaCO_2_, partial pressure of carbon dioxide; PaO_2_, partial pressure of oxygen; Post: post-ECMO/CPB, after cessation of ECMO/CPB; SD: standard deviation.

### Antidote oligonucleotide reverses the effect of the aptamer, similar to protamine reversal of heparin

To assess the drug and antidote’s activity in both groups, we measured the ACT, aPTT, and platelet count of each animal over the course of the experiment.

**Figure 3.**
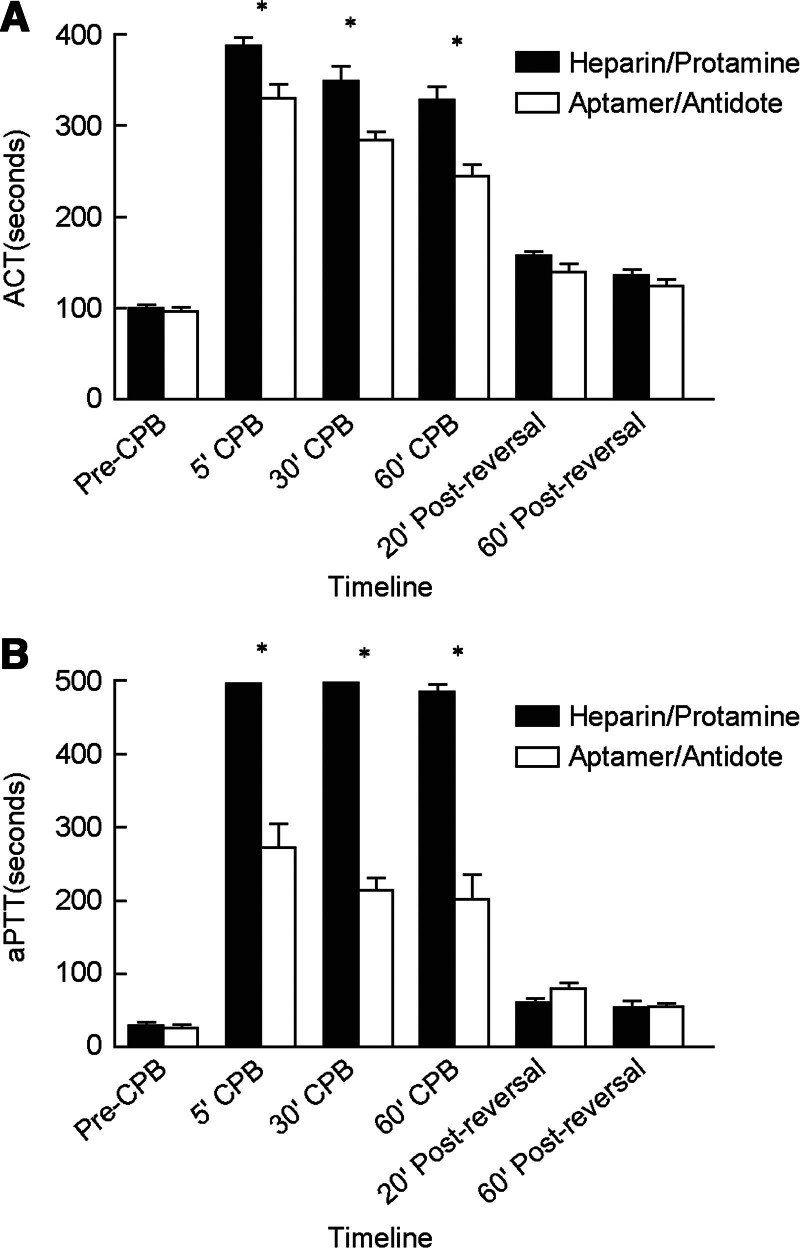
**Heparin- and FIXa aptamer-treated animals showed increases in clotting parameters and equivalent sustained return to baseline after antidote administration. A**, ACT (n = 10). Factor IXa aptamer administration (10 mg/kg, bolus injection) prolongs clotting time measured by ACT. Antidote oligonucleotide (50 mg/kg, bolus injection) efficiently reversed anticoagulation after 60 min of ECMO/CPB. Heparin administration (600 IU/kg, bolus injection) prolongs clotting time measured by ACT. Protamine administration (1 mg/100 IU heparin, bolus injection) efficiently reversed anticoagulation after 60 min of ECMO/CPB; **B**, aPTT (n = 10). Factor IXa aptamer administration (10 mg/kg, bolus injection) prolongs clotting time measured by aPTT. Antidote oligonucleotide (50 mg/kg, bolus injection) efficiently reversed anticoagulation after 60 min of ECMO/CPB. Heparin administration (600 IU/kg, bolus injection) prolongs clotting time measured by aPTT. Protamine administration (1 mg/100 IU heparin, bolus injection) efficiently reversed anticoagulation after 60 min of ECMO/CPB. Solid black bars represent heparin/protamine group, open boxes represent aptamer/antidote group. Error bars represent SD. In animals treated with heparin, the aPTT values during ECMO/CPB exceeded the upper limit of the assay and were noted as 500 s. As no absolute maximum value could be determined at the 5- and 30-min times, no error bars are presented for the heparin samples at these time points. ^*^P < 0.05 compared to heparin group at indicated time points. ACT: activated clotting time; aPTT: activated partial thromboplastin time; CPB: cardiopulmonary bypass; ECMO: extracorporeal membrane oxygenation; FXIa: factor IXa; SD: standard deviation.

In both groups, the ACT was normal before administrating the drug (99 ± 8 in the heparin group and 96 ± 9 seconds in the aptamer group) (Figure 3A). After administration of the anticoagulant drug, a significant rise in ACT was seen in both groups. At the start of ECMO/CPB, the ACT was 389 ± 32 seconds in the heparin group and 335 ± 48 seconds (P = 0.01) in the aptamer group. Neither group received a subsequent dose of anticoagulant during the experiment. At 30 and 60 minutes ECMO/CPB, the ACT remained relatively stable in the heparin- and aptamer-treated animals (349 ± 55 seconds vs. 292 ± 39 seconds, P = 0.03 and 320 ± 54 seconds vs. 254 ± 51 seconds, P = 0.01, respectively) (Figure 3A). After administration of the antidotes, both ACT levels decreased to 130 seconds, which remained stable to the end of experiment.

With respect to the aPTT, after heparin administration, the aPTT measured 500 seconds for most of the ECMO/CPB period. In the aptamer group, the aPTT rose to 260 ± 112 seconds at the start of ECMO/CPB, 240 ± 103 seconds at 30 minutes of ECMO/CPB and 193 ± 112 seconds at the end of ECMO/CPB (P < 0.0001 compared to heparin group during these time points) (Figure 3B). After administration of the antidote, the ACT levels returned to near baseline within 20 minutes of treatment in both the heparin and aptamer group, 60 ± 13 seconds and 79 ± 28 seconds, respectively (P = 0.20), and remained stable to the end of the experiment.

### Improved platelet preservation in aptamer-treated animals

HIT is a known complication of heparin use. Moreover, even in naive patients, heparin administration results in decreased platelet counts^[29–30]^. To assess the effect of each anticoagulant drug on platelet number, platelet count was measured at the beginning of the experiment and again 3 hours after reversal of CPB. The aptamer group showed an improved platelet count at the end of the experiment compared to the heparin group. The platelet or platelet count was measured before drug delivery and again 3 hours post-ECMO/CPB. The thrombocyte count in the heparin group was 729 ± 93 (before ECMO/CPB) and 325 ± 85 × 10^9^/L (post-ECMO/CPB), respectively. In the aptamer group, 530 ± 124 and 247 ± 121 × 10^9^/L platelets were observed. Both groups had a lower post-ECMO/CPB platelet count than pre CPB (time effect, P < 0.001), but the decrease in the heparin group was more pronounced than in the aptamer group (interaction effect, P = 0.01). Thus the change in platelets in the heparin-treated group was −404,000 ± 89,000 platelets/μL and only −288,000 ± 121,000 platelets/μL in the aptamer-treated group (P = 0.03).

### Comparable inflammatory profiles in aptamer-antidote and heparin-protamine groups

**Figure 4.**
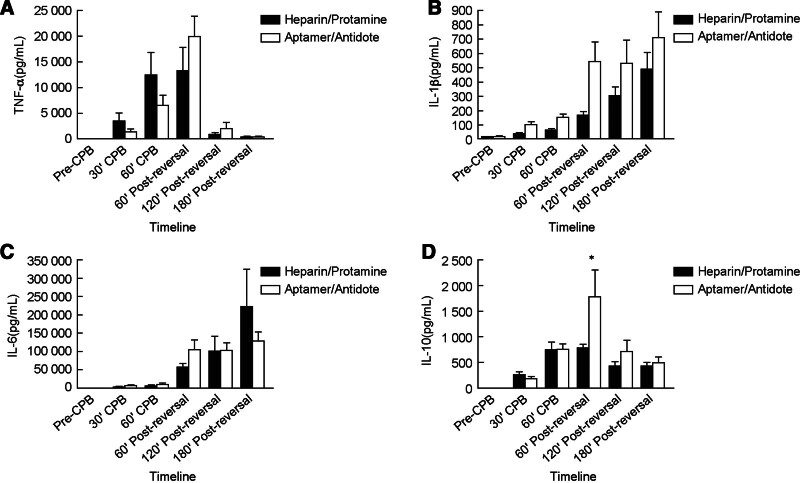
**Heparin- and FIXa aptamer-treated animals showed similar cytokine responses with respect to TNF-α, IL-1β, and IL-6, and a transient, differential response of IL-10 during the course of the experiment that returned to equivalent levels at the end of the experiment. A,** TNF-α (n = 10). Levels in both heparin- and FIXa aptamer-treated groups returned to near baseline 180 min after reversal of ECMO/CPB (P = 0.97); **B**, IL-1β (n = 10). Levels in both heparin- and FIXa aptamer-treated groups increased equivalently throughout experiment (P = 0.34); **C,** IL-6 (n = 10) reflected similar trend as IL-1β, where both heparin- and FIXa aptamer-treated groups increased equivalently throughout experiment (P = 0.47); **D,** For IL-10 (n = 10), a statistically significant difference between the heparin- and aptamer-treated groups (P < 0.05) largely due to a high IL-10 level in the aptamer-treated group 60 min post-reversal ECMO/CPB. The level of IL-10 at the end of the experiment, however, was similar in both groups (P = 0.68).Solid black bars represent heparin/protamine group, open boxes represent aptamer/antidote group. Error bars represent SD. ^*^P < 0.05. CPB: cardiopulmonary bypass; ECMO: extracorporeal membrane oxygenation; IL-1β: interleukin 1-beta; IL-6: interleukin 6; IL-10: interleukin 10; SD: standard deviation; TNF-α: tumor necrosis factor alpha.

To evaluate the inflammatory effects of the heparin-protamine and the FIXa aptamer-antidote, we measured TNF-α, as well as IL-1β, IL-6, and IL-10. The levels of all four markers were below detection at the start of the experiment. The TNF-α levels for the heparin- and aptamer-treated groups peaked 60 minutes (Figure 4A). There was no significant difference between the mean value of 13,266 ± 4,615 pg/mL seen in heparin-treated animals compared to 19,920 ± 4,000 pg/mL seen in aptamer-treated animals (P = 0.70) (Figure 4A). ANOVA across the entire study also reflected no difference between the groups (P = 0.20). In both sets of animals, TNF-α levels returned to near pre-ECMO/CPB levels (P = 0.97).

No significant difference in IL-1β levels was observed between the aptamer- and heparin-treated animals throughout the experiment (P = 0.30) (Figure 4B). In both groups, levels rose significantly by 60 minutes after reversal and continued to rise to the last measured time point, 3 hours after the reversing agent was administered. No significant difference was observed for the peak level of 487 ± 387 pg/mL of IL-1β 3 hours after reversal in the heparin-treated animals compared to 710 ± 582 pg/mL in aptamer-treated animals (P = 0.70) (Figure 4B).

The IL-6 levels followed a similar pattern to the IL-1β data (Figure 4C). In both groups, the levels remained near baseline throughout the ECMO/CPB portion of the experiment. Post-ECMO/CPB, these levels increased and remained elevated to the last measured time point, 3 hours after anticoagulant reversal. No difference was observed between the two groups throughout the experiment (P = 0.47). Also, no difference was observed in the peak levels of 129,892 ± 156,337 pg/mL of IL-6 seen 3 hours after reversal in heparin-treated animals compared to 127,178 ± 85,501 pg/mL in aptamer-treated animals (P = 1.00) (Figure 4C).

The IL-10 inflammatory marker showed a significant difference between the heparin- and aptamer-treated animals throughout the experiment (P = 0.04) (Figure 4D). This is mainly due to the significant increase in IL-10 for the aptamer-treated animals 60 minutes post-reversal (1,782 ± 1,675 pg/mL) compared to the heparin-treated animals (782 ± 253 pg/mL) (P = 0.04). At the end of the experiment, the IL-10 levels were similar for the heparin-treated (491 ± 399 pg/mL) and aptamer-treated animals (430 ± 242 pg/mL) (P = 0.70) (Figure 4D).

### Similar cardiac histopathology in heparin-protamine and aptamer-antidote groups

To determine if differences in cardiac histopathology existed between heparin-protamine-treated animals and aptamer-antidote-treated animals following ECMO/CPB treatment, the hearts of the animals in each study group were harvested and analyzed for the number of lesions and the size of the lesions in the right ventricle (RV), intraventricular septum (IVS) and left ventricle (LV). Interestingly, there was no significant difference in the number of lesions in the RV, IVS, or LV (P = 0.40) (Table 2). The number of lesions in the RV of the heparin-treated animals was 1.3 ± 0.9 lesions compared to the aptamer-treated animals, 1.2 ± 1.1 lesions (P = 0.80). This result also held for the IVS, with the heparin-treated group having 0.6 ± 0.5 lesions vs. the aptamer-treated group having 0.5 ± 0.5 (P = 0.70) and LV with the heparin-treated group having 2.1 ± 1.0 lesions vs. the aptamer-treated group having 2.9 ± 0.9 (P = 0.10) (Table 2).

**Table 2.** Histopathological data: number and size of areas of myocardial contraction band necrosis in the two groups

	RV	IVS	LV
Number of MCBN			
Heparin	1.3 (0.9)	0.6 (0.5)	2.1 (1.0)
Aptamer	1.2 (1.1)	0.5 (0.5)	2.9 (0.9)
Area size, mm^2^			
Heparin	1.6 (1.6)	0.6 (0.8)	3.6 (3.0)
Aptamer	1.1 (1.4)	0.4 (0.9)	5.2 (2.5)

Data are presented as mean ± SD.

IVS: intraventricular septum; LV: left ventricle; MCBN: myocardial contraction band necrosis; RV: right ventricle; SD: standard deviation.

Also, no significant difference in the size of the lesions was observed between the heparin- and aptamer-treated animals (P = 0.90). The aggregate size of the lesions in the RV for the heparin-treated animals was 1.6 ± 1.6 mm compared to 1.1 ± 1.4 mm for the aptamer-treated group (P = 0.30). The IVS and LV revealed similar results with IVS lesions in the heparin-treated group being 0.6 ± 0.8 mm vs. 0.4 ± 0.9 mm in the aptamer-treated group (P = 0.50). The LV revealed 3.6 ± 3.0 mm and 5.2 ± 2.5 in the heparin- and aptamer-treated groups, respectively (P = 0.60) (Table 2).

### Improved cardiac function in aptamer-antidote group vs. heparin-protamine by echocardiography

**Figure 5.**
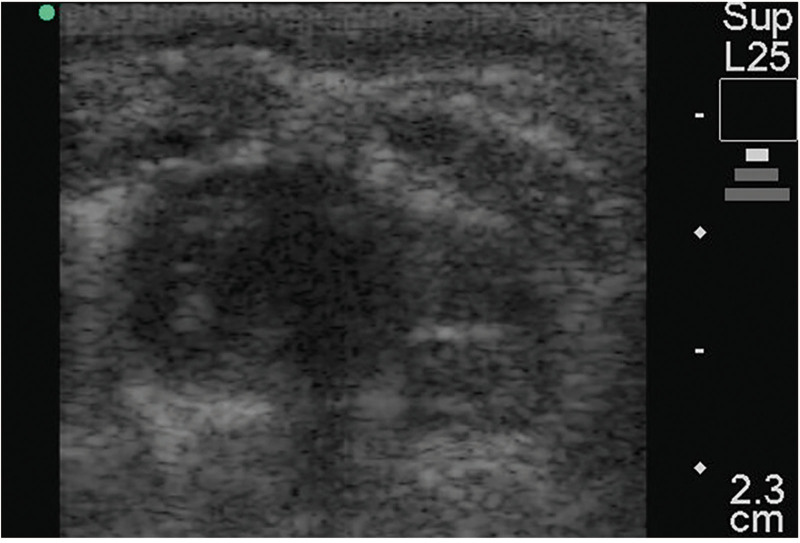
**Echocardiography analysis.** Three hours after the cessation of ECMO/CPB, an estimate of the SF was obtained in all rats using ultrasonography. A transthoracic cross-sectional view of the left ventricle was obtained at papillary level. The EDD and ESD were measured and the SF calculated (EDD − ESD/EDD × 100) for both groups of rats. Rat heart rates remained > 200 bpm during the analyses. The SF was 42% ± 8% and 60% ±16% in heparin group and FIXa aptamer group, respectively (P = 0.01). bpm: beats per minute; ECMO/CPB: extracorporeal membrane oxygenation/cardiopulmonary bypass; EDD: end-diastolic diameter; ESD: end-systolic diameter; FXIa: factor IXa; SF: shortening fraction.

In addition to assessing the MAP, we wanted a real-time measurement of vessel diameter to correlate with the improved cardiac output in the aptamer-treated animals compared to the heparin-treated animals. An ultrasound image was obtained of the left ventricle at the end of the experiment in the heparin- and aptamer-treated animals, and a SF was calculated. The aptamer-treated group showed improved cardiac function compared to the heparin-treated animals with an SF of 60% ± 16% vs. 42% ± 8% (P = 0.01) (Figure 5).

## DISCUSSION

Our results demonstrate that the FIXa aptamer and antidote can successfully replace heparin and protamine in a rat ECMO circuit designed to mimic a CPB circuit. In both groups, the circuit remained patent and without evidence of clot formation at the end of the study. The ACT and aPTT values in the aptamer-treated group were lower than those of heparin. An obvious concern with the lower measured clotting times is the suggestion that there may be more thrombosis in the aptamer-treated group. The inspection of the circuits in each group provided a macromeasurement that there was no increase in thrombosis. Interestingly, while both ACT and aPTT are standard-practice tools in evaluating anticoagulation and are both sensitive to FIXa inhibition, a significant amount of thrombin generation and subsequent inflammation still occurs^[1,31]^. However, we previously reported a lower thrombin generation profile in FIXa aptamer-treated animals compared to those that received heparin in a neonatal swine model of CPB^[14]^. Similarly, we have observed that a pegylated version of the FIXa aptamer can maintain a neonatal swine ECMO circuit clot-free for 12 hours^[32]^, even though we also observed that this aptamer could not maintain an *ex vivo* circuit clot-free when utilizing adult human blood^[33]^. Nevertheless, these studies on adult rats indicate that a cholesterol-modified version of the FIXa aptamer is potent enough in adult blood to maintain an ECMO/CPB circuit clot-free. Moreover, our results suggest that circulation of whole blood through an *ex vivo* oxygenator circuit, without also circulating the blood through an animal, requires even higher levels of anticoagulation than animal models of ECMO/CPB^[33–34]^.

Since an intimate relationship exists between thrombosis and inflammation, we measured inflammatory markers TNF-α, IL-1, and IL-6 that are known to be elevated in CPB surgery^[35–36]^. All three cytokines showed similar levels in the aptamer-antidote treated group compared to the heparin-protamine treated rats. Only IL-10 showed some increases mid-experiment that returned to similar levels in the heparin-treated animals by the end of the experiment. We found the cytokine data interesting, as previously we had shown an improved inflammatory profile in FIXa aptamer-treated pigs. This difference could be due to the animal used in the study, the model, or both. The previous survey was done in a neonatal swine where the bypass was done in the right atrium and aorta. The heart was arrested for 30 minutes and underwent cardioplegia^[14]^. The rat model used in this experiment underwent bypass by the right atrium and the tail artery, with no way to arrest the heart and perform cold cardioplegia. It would be interesting to evaluate the relative cytokine levels in a more robust rat CPB model where the heart is arrested, though this is experimentally challenging. Finally, in this experiment, the FIXa aptamer dose was 5 mg/kg, which was 10-fold higher than that used in the porcine bypass experiments^[14]^. Species difference could account for this finding; however, it is also possible that the age of the animals used in the models could have played a role. Finally, we recently observed that the FIXa aptamer is more potent in pig plasma as compared to rat plasma as measured by aPTT^[37]^. As mentioned earlier, we used neonatal piglets in our previous studies, while this study utilized adult rats. Regardless, these observations are important as the FIXa aptamer moves forward in clinical development; pharmacokinetic differences between children and adults will be important to understand in establishing dosing guidelines.

The FIXa aptamer and antidote offer potential advantages over UFH and protamine by improving platelet count and greater cardiac output as measured by flow probe and ultrasound. HIT is a well-recognized complication associated with using heparin. Typically, a patient is given heparin, which binds to the platelet surface to PF4. Antibodies recognize the heparin–PF4 complex and neutralize the platelet that carries the complex. This interaction can lead to thrombocytopenia and thrombosis. Many clinicians will hesitate to re-use heparin, if after first exposure, there is a significant decrease in platelet count^[38]^. Our studies revealed a relatively stable platelet count postoperatively in the aptamer-treated animals compared to the heparin-treated animals. Even in the absence of known HIT, the FIXa aptamer may confer a margin of safety in patients who are relatively thrombocytopenic preoperatively.

A clear advantage of using heparin in CPB is that protamine is a readily available reversing agent should untoward complications arise that necessitate cessation of anticoagulation. Unfortunately, using protamine is not without risk. Systemic hypotension and pulmonary hypertension can limit its use in CPB surgery^[39]^. Moreover, hypersensitivity reactions to protamine sulfate can occur and even be life-threatening^[40–41]^. The antidote oligonucleotide is effective in quickly reversing the anti-FIXa aptamer activity. Furthermore, the improved MAP and SF suggest that this approach might be safer and more effective than heparin and protamine in certain patients with underlying cardiovascular disease. The improved SF is remarkable. More research is needed to point out the true mechanism. However, as inflammation in both groups appears equal, the reduced SF does not appear to be due to an inflammatory response or release of cardiodepressive factors. A more likely possible explanation could be that the effect observed is the reflection of the direct cardiac-depressive effect of protamine, which is not present when the antidote oligonucleotide is utilized to reverse the anticoagulant effect of the aptamer. This depressive effect by protamine has been described before in isolated myocytes and in ischemic rat hearts in a Langendorff preparation^[42–43]^. In these articles, the authors showed that isolated rat hearts subjected to ischemia are more vulnerable to the effects of protamine than are non-ischemic hearts. They noted reduced myocardial performance, reduced LV pressures, and a decrease in coronary flow after protamine administration in ischemic hearts. Our results may reflect that observation.

These experiments show that the FIXa aptamer and antidote are an effective reversible anticoagulant in a rat CPB model of ECMO/cardiac surgery. They also demonstrate that the aptamer and antidote oligonucleotide approach has potential advantages over heparin and protamine by preserving platelet count and maintaining a favorable cardiac profile over the course of the experiment. This anticoagulant drug–antidote pair may provide an attractive alternative to patients who cannot use heparin and protamine secondary to HIT or allergy to heparin. Finally, these studies indicate that a cholesterol-modified version of the FIXa aptamer can maintain an ECMO/CPB circuit in an adult animal clot-free. As mentioned earlier, a pegylated version of this aptamer is also able to maintain a neonatal swine ECMO circuit clot-free. Unfortunately however, though the pegylated aptamer was at least as effective as UFH in limiting thrombosis in a phase 2 clinical study in patients undergoing percutaneous coronary intervention^[44]^, rare patients with high levels of anti-polyethylene glycol (PEG) antibodies experienced severe allergic reactions to the pegylated drug^[45]^, a result confirmed in a larger phase 3 study^[46]^. Subsequently, it was also observed that such anti-PEG antibodies can also limit the anticoagulant activity of a pegylated aptamer^[47]^. Therefore, the clinical development of a cholesterol-modified version of the FIXa aptamer instead of a pegylated version appears to be warranted.

### Limitation

Limitations of this study include that all studies were performed using either in vitro measures of blood coagulation or in a small animal model of ECMO-CPB. As this murine model does not allow stoppage of the heart, it may not totally reflect the hemostatic and thrombotic effects encountered in humans undergoing cardiopulmonary bypass. Moreover the ECMO circuit was maintained for a relatively short period of time, a couple hours, compared to clinical situations requiring ECMO for days or even weeks. Thus additional studies should be performed on large animals such as swine that are amenable to ECMO for 12-24 hours as murine models are not. Finally as the aptamer was generated against the human factor IXa protein and not the rat factor IXa protein, differences in binding of the aptamer to the human versus the rat factor may alter the potency of the inhibitor in humans subject to ECMO as compared to rats.

## CONCLUSIONS

A nuclease resistant, cholesterol modified ribonucleic acid (RNA) aptamer that binds and inhibits human factor IXa cross reacts with rat factor IXa. The aptamer can effectively anticoagulant rats when administered intravenously and is potent enough to maintain a ECMO circuit clot free when a is rat subject extracorporeal membrane oxygenation. Moreover the aptamer is rapidly reversible with an antidote oligonucleotide that is complementary to a portion of the aptamer. Thus this aptamer-antidote pair represents an approach to effectively control blood coagulation in highly prothrombotic settings such as ECMO.

## FUNDING

This work was funded in part by a grant from the NIH (P01 HL139420) to BAS.

## AUTHOR CONTRIBUTIONS

SMN devised and conducted experiments, interpreted data, and wrote manuscript; FdL devised and conducted experiments, interpreted data, and wrote manuscript; GAP devised and conducted experiments, interpreted data, and wrote manuscript; and BAS devised experiments, interpreted data, secured funding, and wrote manuscript.

## CONFLICTS OF INTEREST STATEMENT

Duke University has submitted patent applications on anticoagulant aptamers and Drs. Shahid M. Nimjee, George A. Pitoc, and Bruce A. Sullenger are inventors on such applications.

## ACKNOWLEDGMENT

We would like to thank G. Burkhard Mackensen for discussion and advice on experimental design.

## DATA SHARING STATEMENT

All data that supported the findings of this study are included in this article and all inquiries about data and access to such information should be directed to Dr. Bruce A. Sullenger (bruce.sullenger@duke.edu) or Dr. Shahid M. Nimjee (shahid.nimjee@osumc.edu).
